# 常压质谱用于脂质识别的研究进展

**DOI:** 10.3724/SP.J.1123.2024.06007

**Published:** 2025-01-08

**Authors:** Xiaorong WANG, Yiyan YIN, Jin OUYANG, Na NA

**Affiliations:** 1.北京师范大学化学学院,放射性药物化学教育部重点实验室, 北京 100875; 1. Key Laboratory of Radiopharmaceuticals, Ministry of Education, College of Chemistry, Beijing Normal University, Beijing 100875, China; 2.北京师范大学文理学院化学系, 广东 珠海 519087; 2. Department of Chemistry, College of Arts and Sciences, Beijing Normal University, Zhuhai 519087, China

**Keywords:** 常压质谱, 离子化技术, 脂质, 脂质精细结构, 串联质谱, ambient mass spectrometry (AMS), ionization techniques, lipid, lipid fine structure, tandem mass spectrometry (MS/MS)

## Abstract

脂质是生物体的重要组成成分,参与细胞膜流动、神经递质传递和运输以及能量供应等多个过程。研究表明,癌细胞为了适应不断变化的生物微环境和快速增殖的需求,其脂质代谢过程不同于正常细胞。因而,对脂质组分进行快速检测、识别及监测研究,对了解生命过程、监测诊疗过程变化、提升诊疗效率等具有重要意义。质谱是直接获取分子结构最有效的手段之一,在生物分子及脂质鉴定中具有独特优势。近年来,常压质谱技术(ambient mass spectrometry, AMS)不断涌现,该技术无需样品预处理,为直接快速脂质识别及监测提供了有效手段。软电离技术的不断发展也为复杂多样的脂质分子检测提供了广阔的发展空间。电喷雾离子化(ESI)作为主要的软电离技术之一,易于离子化中高极性的生物样品,能够满足生物体内大多数脂质的检测需求。因此,研究人员基于ESI技术,开展了广泛的癌症脂质代谢研究,实现了不同脂质的鉴定和相对定量研究。由于脂质存在丰富的异构体,人们又创新性地将各种化学衍生法和其他技术与AMS结合起来,实现了对复杂脂质结构异构体的准确识别和相对定量研究。基于此,本文综述了近5年用于脂质检测的主要常压质谱技术及应用进展,并汇总了质谱解析脂质精细结构的典型策略。

## Abstract (en)

Lipids are indispensable components of living organisms and play pivotal roles in cell-membrane fluidity, energy provision, and neurotransmitter transmission and transport. Lipids can act as potential biomarkers of diseases given their abilities to indicate cell-growth status. For example, the lipid-metabolism processes of cancer cells are distinct from those of normal cells owing to their rapid proliferation and adaptation to ever-changing biological environments. As a result, the ability to rapidly detect, identify, and monitor lipid components is critical for tracking life-related processes and may enhance cancer diagnosis and treatment efficacy. Mass spectrometry (MS) is regarded to be among the most efficient methods for directly obtaining molecular-structural information, and is distinctly advantageous for identifying lipids. Recent years have witnessed the emergence of ambient mass spectrometry (AMS), which enables direct analyte sampling and ionization without the need for sample preprocessing. These characteristics endow AMS with special advantages for identifying and monitoring lipids. Furthermore, the ongoing development of soft ionization technologies has led to the widespread use of AMS for the detection of complex and diverse lipid molecules. Electrospray ionization (ESI) is a gentle ionization method that can be used to detect medium-to-high-polarity compounds and provide detailed chemical information for lipids by producing a fine mist of charged droplets from a liquid sample. Consequently, a series of ESI-based ionization methods have been developed for fabricating different AMS systems capable of rapidly detecting lipids in a simple manner. For example, desorption electrospray ionization (DESI) is among the most extensively employed ambient ionization techniques, and has been used to detect a wide range of samples, including solids, liquids, and gases. DESI involves spraying a charged solvent onto the surface of a sample, after which the solvent is desorbed, the analyte is ionized, and the generated ions are transferred to the detector of the mass spectrometer via a gas plume. DESI can easily and precisely regulate the sampling space, thereby offering a highly effective approach for the in-situ detection of lipids from tissue samples. Additionally, single-cell lipid analysis is limited by small cell volumes, complex cellular matrices, and minimal absolute amounts of analyte. Common detection methods for single cells include flow cytometry and fluorescence microscopy, both of which require fluorescent labeling to detect specific target molecules, which limits detection selectivity and reproducibility to some extent. ESI-based single-cell mass spectrometry has emerged as a more-effective method for detecting cellular lipids owing to advantages that include high sensitivity, low sample consumption, high throughput, and multiple-detection capabilities. Moreover, lipid chemical diversity poses a significant challenge for determining structural details. Therefore, AMS-based lipid detection has been augmented with a series of chemical-treatment methods that provide more-comprehensive structural information for lipids. For example, diverse gas-phase dissociation techniques have been used to discriminate between lipid C=C-bond isomers and their *sn*-positions. Strategies that involve chemically modifying specific target C=C bonds prior to MS detection have also been employed. For example, the Paternò-Büchi (P-B) photochemical reaction oxidizes C=C bonds in unsaturated lipids to form oxetane structures, C=C bonds can be epoxidized to form the corresponding oxaziridines, the N-H aziridination reaction converts C=C bonds into aziridines, and the ^1^ΔO_2_ ene reaction adds an OOH group to a C=C bond. In this review, we discuss various environmental ionization techniques for lipid AMS developed over the past five years, with an emphasis on typical chemical strategies used to analyze lipid fine structures. Obtaining a high-coverage, high-sensitivity lipid-detection platform based on AMS remains challenging and requires further in-depth studies despite significant improvements in lipid MS-based detection techniques.

脂质是一类疏水性或两亲性小分子,可以通过硫酯(脂肪酸、聚酮类等)的碳负离子缩合和(或)异戊二烯(异戊二烯醇、甾醇等)的碳正离子缩合等过程产生。基于化学结构的差异,脂质一般可以分为脂肪酸(fatty acid, FA)、甘油酯(glycerolipids, GL)、甘油磷脂(glycerophospholipids, GP)、鞘脂(sphingolipids, SP)、甾醇脂质(sterol lipids, ST)、糖脂(glycolipids, SL)和聚酮(polyketides, PK)(图1)^[1]^。

**图1:**
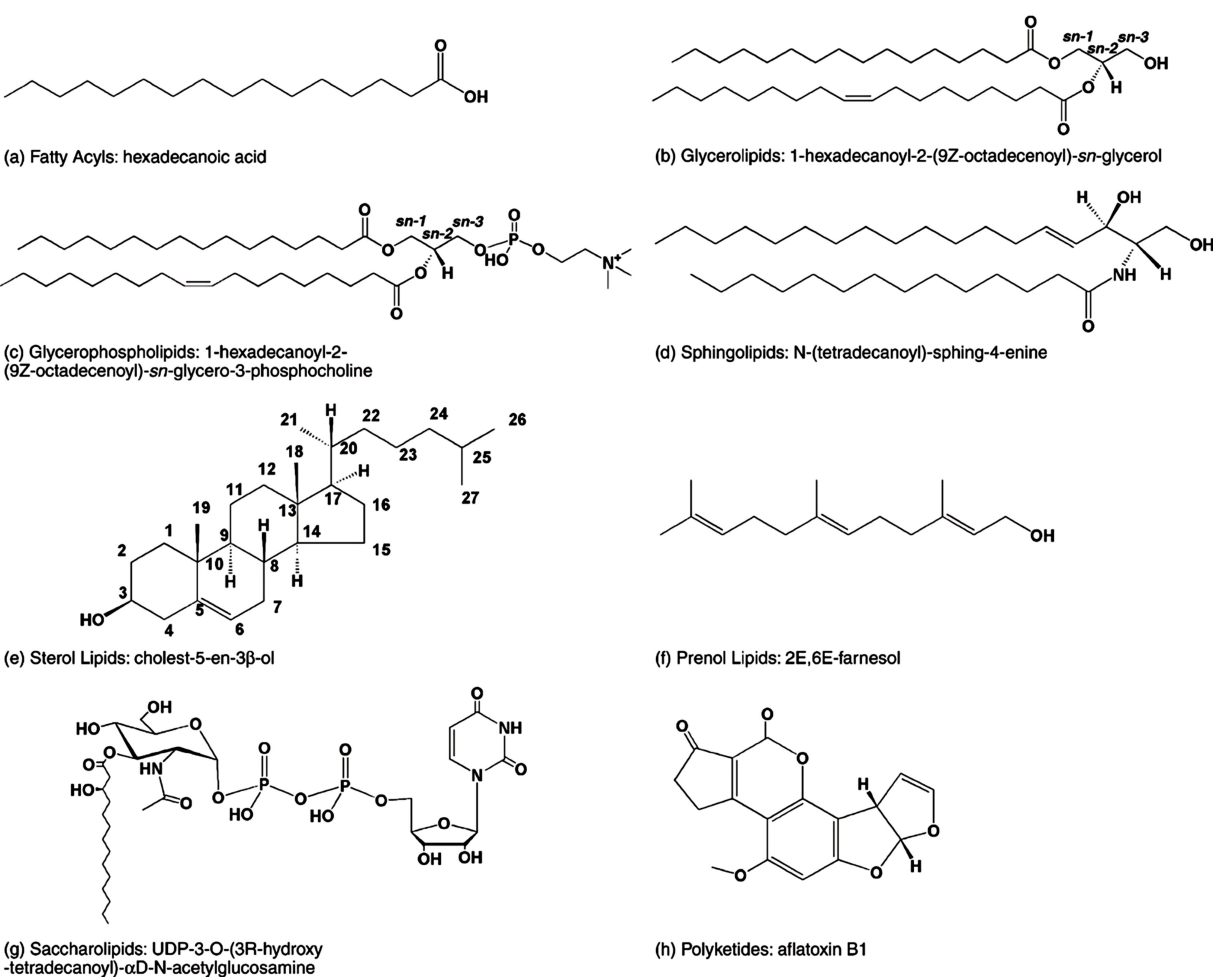
脂质的代表性结构^[1]^

对于脂质组成和精细结构的研究可以帮助人们更多地认识脂质在膜结构、能量产生和信号通路中发挥的作用。大量的研究也表明,癌细胞因为要满足不断增殖的能量需求和适应不断变化的微环境,其各种代谢过程(包括脂质代谢途径)与正常细胞具有显著差异^[2⇓⇓⇓⇓-7]^。因此,我们可以通过脂质代谢的研究来判断人体是否罹患疾病,例如乳腺癌、胃肠道癌以及心血管疾病等。更进一步,对脂质的深入研究还能为癌症诊断鉴别提供信息^[8]^,有助于手术切除边界的评估^[9]^,并为多种疾病的分子病理机制^[10]^研究提供有价值的信息。这些需求极大地推进了脂质组学的发展。

脂质组学的主要研究任务是对脂质进行定性和定量研究,分析脂质空间分布和动态,以及脂质在生物过程中发挥的作用^[11⇓⇓⇓⇓-16]^。在脂质组学研究中,质谱(MS)由于具有高灵敏度和高通量的特征,能够为研究人员准确提供复杂生物体系中脂质分子的结构和数量的丰富信息,受到了广泛关注^[17]^。质谱是一种基于分子离子和特征碎片离子的质荷比进行化合物鉴定和定量分析的技术,在脂质研究中占有重要的地位。其中,常压质谱(ambient mass spectrometry, AMS)是近年来新兴的一类快速检测质谱技术,其无需样品预处理即可从常压环境中直接抽提和离子化物质^[18,19]^,能够对固、液、气态的样品进行快速、实时、高通量的原位分析^[20⇓⇓-23]^。基于不同的样品离子化方式,AMS的离子源主要可以分为电喷雾电离(electrospray ionization, ESI)和大气压化学电离(atmospheric pressure chemical ionization, APCI)两类;其中,ESI易于分析中高极性分子,而APCI易于分析中低极性的样品。针对脂质检测的不同需求,人们构建了不同种类的离子化技术,并开展了广泛的应用研究。

## 1 用于脂质检测的AMS方法总述

由于生物体内的脂质主要是中高极性的物质,因此,ESI技术能够满足大多数生物样品中脂质分子的检测需求,而APCI技术只能检测部分中性脂质分子,如甘油三酯和甾醇等。因此,为了确保检测到细胞内完整的脂质相关的信息,ESI是生物体内脂质检测研究的首选方法。在基于ESI的AMS检测中,首先从固体或液体样品中抽提待测物质并生成带电液滴,继而蒸发带电液滴中的溶剂,引发库仑爆炸,从而获得了带电离子,最后,所生成的离子被输送进质谱检测器进行检测。该离子化过程包括3种形式:在正离子和负离子模式下形成加合物;由盐直接产生完整的阳离子和阴离子;在负离子模式下去质子化。根据不同的解吸附或离子化方法差异,用于脂质检测的主要AMS技术包括电喷雾电离质谱(electrospray ionization mass spectrometry, ESI-MS)和解吸电喷雾电离质谱(desorption electrospray ionization mass spectrometry, DESI-MS)。其中,Cooks等^[24]^在2004年首次提出DESI适用于分析极性至中极性脂质物质,如FA、GL、GP、SP、SL等。该技术利用带电溶剂喷雾撞击样品表面,直接离子化样品表面分子,避免了复杂的样品制备过程,在快速检测中展现出巨大的应用前景。如图2^[25]^所示, DESI的离子化机制是带电喷雾撞击样品表面,同时产生薄膜,并发生微萃取过程;随后溶解了分析物的微滴从薄膜溅出;最后,基于电喷雾原理,液滴中的样品被离子化,随后进入质谱检测器进行检测。

**图2:**
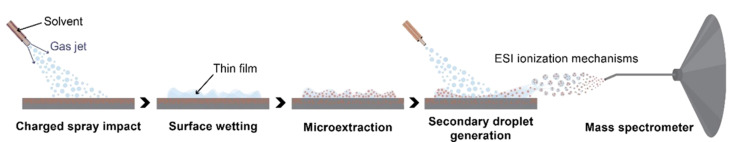
DESI的离子化机制:液滴提取^[25]^

由于脂质分子种类庞大、结构复杂、异构体多且难分离,因此,建立更多的常压离子化质谱技术用于脂质精细结构的快速鉴定具有重要意义,也将为基于脂质的生物诊疗技术的发展奠定基础。串联质谱法(tandem mass spectrometry, MS/MS)首先选择并破裂母离子,随后对产生的碎片离子进行第二次质谱分析,以获取待测物质的质荷比信息。碰撞诱导解离(collision-induced dissociation, CID)是一种软电离技术,通过母离子与其他分子发生碰撞来碎裂母离子,是MS/MS实现母离子碎裂过程中的一个重要步骤。常规MS/MS技术可以区分脂质的头基和脂肪酸链,但很多情况下这类脂质并不产生特征碎片离子,因此无法有效提供C=C键位置、甲基支链等信息。为了解决这一问题,研究人员提出了两种脂质结构鉴定的途径。第一种途径是使用不同的气相解离方法,实现脂质分子C=C处或周围的碎裂,包括电荷远程碎裂^[26]^、臭氧诱导解离(OzID)^[27]^、自由基定向解离^[28]^以及紫外光解离(UVPD)^[29]^等。其中,OzID^[30⇓-32]^和UVPD^[29]^在成功定位脂质C=C位置的同时,还能够区分脂质中的*sn*-位置。第二种途径是在MS分析之前对C=C进行特异性化学衍生,包括Paternò-Büchi (P-B)反应^[33]^、环氧化^[34]^、NH氮丙啶化^[35]^和^1^ΔO_2_ ene反应^[36]^等,对生物样品中不饱和脂质的C=C键进行衍生化,继而通过CID裂解获得特征碎片离子,从而定位不饱和脂质中C=C的位置。

此外,基于MS的脂质组学分析在很多领域如医学、生物学、食品等应用广泛。例如,AMS可以提供食物中的脂质成分^[37]^或饮食干预后生物体中脂质代谢变化信息^[38]^。Janssen等^[39]^基于MS方法选择性检测了食品中的氧化脂质和非氧化脂质,有助于后续对低丰度氧化三酰基甘油的快速检测。Fornal等^[40]^应用MS鉴定了小众食用油(如亚麻荠、亚麻、大麻种子油)的综合脂质谱,为食品的产品认证和掺假检测提供了有力工具,有助于评估脂质食品的安全问题。

本文将首先介绍不同ESI技术应用于不同类型生物样品(如组织、生物流体和细胞)的脂质结构解析的研究进展,继而逐一讨论各种化学衍生法在脂质精细结构解析中的应用。最后,简要概述其他AMS识别脂质异构体的相关应用。

## 2 基于ESI技术的脂质识别研究

### 2.1 ESI-MS

ESI作为一种“软”电离技术,适用于检测中高极性分子,是识别脂质等生物分子最常用的离子化方法之一^[41⇓-43]^。基于ESI-MS的脂质检测和监测能够提供生物诊疗过程中的脂质结构变化信息^[44]^,有助于生物分析^[45]^和临床诊断。如Na等^[46]^应用ESI-MS研究了生物体内最丰富的脂质之一——亚油酸(linoleic acid, LA),并成功监测了脂质过氧化过程中重要物种的动态变化。如图3所示,使用ESI-MS观察到了LA被光催化后的氧化产物和过氧化产物,并对相关离子的质谱信号随时间的变化规律进行了分析,阐述了·OH引发脂质过氧化(lipid peroxidation, LPO)过程的机制。此外,Saghatelian等^[47]^也借助该技术研究了野生型小鼠脂肪组织中的脂质提取物,成功检测了各种内源性羟基脂肪酸支链脂肪酸酯(FAHFAs)。该课题组通过观察不同片段对应的特征磷脂离子,将来自脂肪组织中的离子与标准样品的谱图对照分析,总结出了含有FAHFA的三酰基甘油(FAHFA-TGs)和胆固醇酯(FAHFA-CEs)的碎片化规律,因而鉴定出了新的内源性脂类,揭示了脂质代谢的新途径,为治疗糖尿病和炎症性疾病提供了新思路。此外,Ding等^[48]^采用液相色谱-电喷雾串联质谱法(LC-ESI-MS/MS)分析了分别来自子痫前期患者和健康对照者的胎盘脂质样本,发现患者样本中的不饱和甘油三酯、磷脂酰胆碱、鞘磷脂和磷脂酰丝氨酸的丰度高于对照组的,并根据通路分析判断患者的脂质失调与甘油磷脂代谢密切相关,为确定患者胎盘功能障碍的发病机制和发现潜在的治疗靶点提供了帮助。除此之外,Ahrends等^[49]^将生物惰性反相液相色谱与MS联用对生物样品中的脂质进行分析,能够在20 min内一次性对388种脂质进行灵敏、稳定的监测,成功量化了人血浆中307种内源性脂质分子。

**图3:**
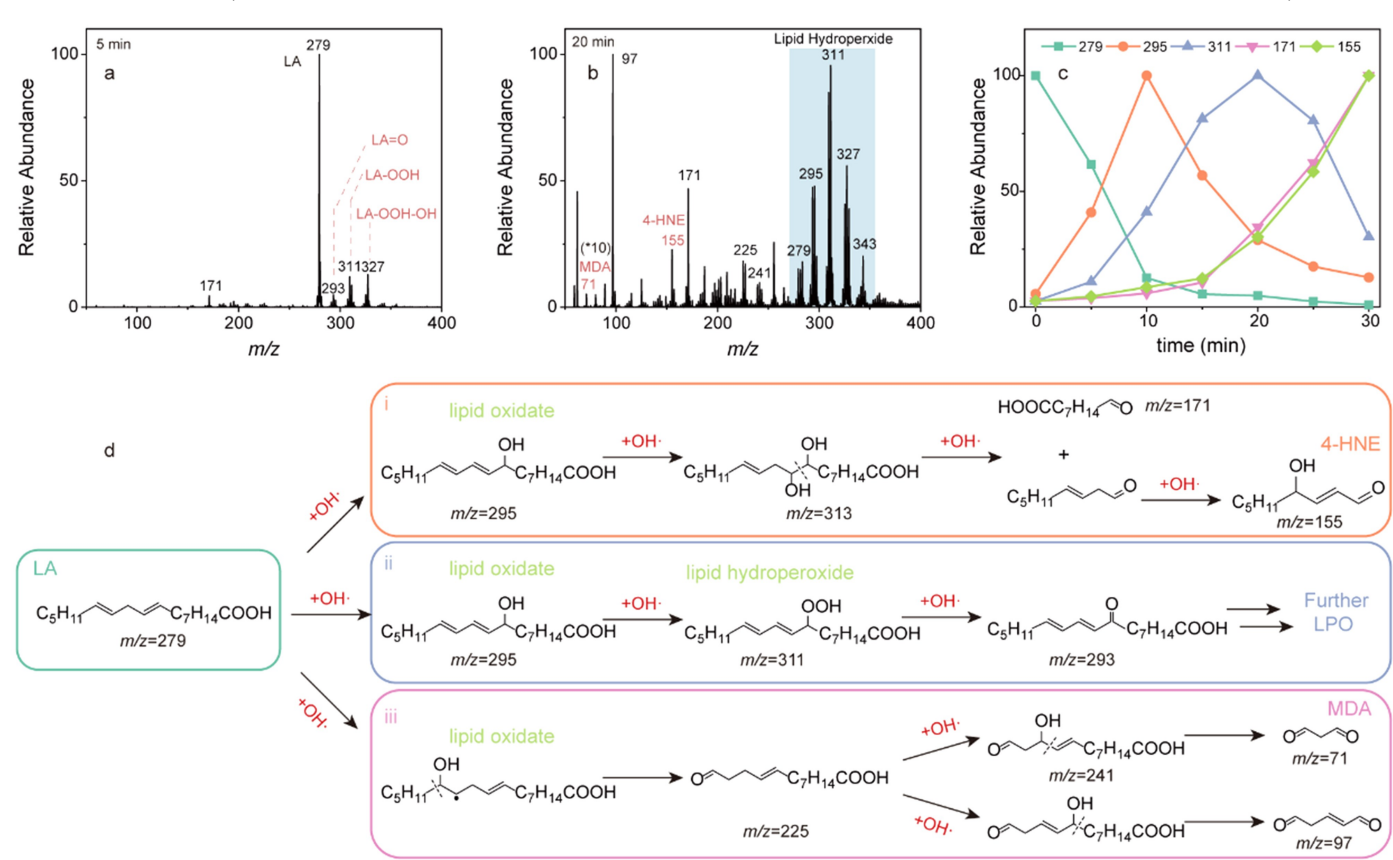
光催化LA过程中脂质过氧化的MS图,LPO过程中相关离子质谱信号的时间依赖及LPO过程的机理阐述^[46]^

此外,AMS技术也是单细胞代谢物分析的有力工具^[50⇓⇓⇓⇓-55]^。单细胞分析的最大挑战在于细胞体积小,细胞基质复杂且分析物绝对量非常少。迄今为止,荧光显微镜^[56]^、单细胞转录^[57]^、流式细胞术^[58]^、拉曼光谱^[59]^、毛细管电泳^[60]^等技术已成功应用于单细胞分析领域。然而这些方法存在很多局限,如选择性低、重复性差、灵敏度低等。而基于ESI技术的单细胞质谱^[61⇓-63]^能够克服单细胞样品量少的局限,在无标记的情况下,同时分析细胞中多种脂质。如Zhang等^[64]^采用ESI-MS实现了数百种单细胞代谢物的高通量(每分钟约38个细胞)无标记分析,相关测试均在QE-Orbitrap质谱仪上进行,质谱相关参数包括:毛细管温度为320 ℃、分辨率为35000、自动增益控制目标值为10^6^以及最大进样时间为10 ms,在细胞注射、代谢物提取和离子化时,以1 μL/min流速提取细胞悬液,在正离子模式下,鞘液为掺杂1%甲酸的甲醇溶液,外加+2.3 kV的高压;在负离子模式下,鞘液为掺杂1%氨水的甲醇溶液,外加-2.3 kV的电压。Ma和Ouyang等^[65]^将电迁移与液滴辅助电喷雾电离(droplet-assisted electrospray ionization, DAESI)相结合用于单细胞质谱分析。

如图4a所示,首先用戊二醛固定细胞,防止细胞在无机盐溶液中裂解。经处理过后的单细胞与2-乙酰吡啶发生P-B反应后,能够通过MS/MS定位其中脂质的C=C异构体。质谱在进行单细胞分析时,如图4b所示,会取适量用水稀释的细胞悬浮液滴在拉尖毛细管上进行纳升电喷雾离子化(nano-ESI),并外加-1.3 kV的直流电压。待nano-ESI尖端溶剂蒸发后,会继续滴加适量含1%甲酸的甲醇-乙腈(1∶1, v/v)辅助溶液,使用DAESI进行单细胞质谱分析。借助该分析平台,成功在单个哺乳动物细胞(如人乳腺癌细胞MDA-MB-231)中检测到多种高丰度的卵磷脂(PCs)。

**图4:**
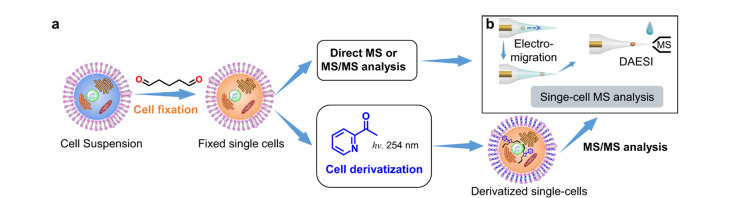
基于电迁移与液滴辅助电喷雾离子化的单细胞分析示意图^[65]^

ESI-MS用于研究脂质的优势在于其灵敏度高、重现性好且能与液相色谱兼容,现已广泛应用于脂质组学研究。但ESI-MS由于其进样特点,基质效应和信号抑制现象明显,还需要进一步改进。

### 2.2 DESI-MS

DESI是目前广泛使用的常压离子化技术,已应用于包括大脑、膀胱、前列腺、脊髓和肾脏等不同类型组织的检测^[66⇓-68]^。DESI的解吸和离子化特点有利于控制采样空间,即高速喷雾液滴可以准确到达样品表面上特定的位置,这是DESI能够作为一种成像技术的关键。2005年,Cooks等^[69]^首次用DESI-MS对生物组织中的磷脂进行了分析。该方法不需要对组织切片进行预处理,即可检测小鼠胰腺、大鼠脑和转移性人肝腺癌组织中脂质的分布。在正离子模式下,DESI-MS检测小鼠胰腺组织时,以含1%醋酸的甲醇-水(1∶1, v/v)为喷雾溶液,流速为3 μL/min,雾化N_2_气压为7 bar。后续工作中,Cooks等^[70]^又将DESI-MS应用于术中切除的脑胶质瘤组织涂片的脂质分析,为病理状态评估提供了有效数据。此外,Laskin等^[71]^搭建了纳米喷雾解吸电喷雾电离质谱(nano-DESI)分析平台,实现了对小鼠子宫组织切片中脂质和代谢物的定量成像。同时获取了具有相同长度酰基链、双键数量不同的几种脂质在组织中空间分布信息。因此,DESI可以通过收集组织切片脂质的质谱数据,结合脂质和代谢物的分析,帮助区分癌症与正常组织。

## 3 基于AMS识别脂质C=C位置异构体

脂质具有丰富的结构信息,包括脂质类别、脂肪酰基/烷基组成以及脂质的*sn*-位置异构、脂质中碳碳双键(C=C)位置及其顺反异构等。不饱和脂质占哺乳动物细胞总脂质比例很大,其生物功能与C=C位置密切相关。因此,准确鉴定和识别脂质结构对于理解脂质相关的生命过程具有重要意义。典型的C=C特异性化学衍生或反应联合MS/MS精确鉴定脂质异构体的方法概述如下。

### 3.1 基于P-B反应的脂质C=C位置异构体鉴定

P-B反应是羰基化合物与烯烃的[2+2]光化学反应,即在紫外光照射下,醛和酮的羰基被活化为双自由基^[72]^并与烯烃中的C=C发生化学反应。Xia等^[33]^于2014年首次将P-B反应和nano-ESI-MS/MS相结合,对脂质中的双键进行定位。该新方法的原理是在紫外线照射下,脂质和丙酮发生P-B反应,再通过碰撞活化,进而对碎片离子进行分析,确定脂质双键位置。如图5所示,在距离质谱采样口0.5~1.0 cm处搭建nano-ESI源,紫外灯在距离nano-ESI针尖1.5~2.5 cm处进行照射,以含1% NH_4_OH的丙酮-水(50∶50, v/v)溶解脂质,外加±1.5~1.8 kV的电压。Xia和Ouyang等^[73]^创新性地将P-B反应在线C=C衍生化搭载到脂质组学研究最常用的液相色谱-串联质谱(LC-MS/MS)平台上,开发出了大规模的脂质分析平台;借助该平台成功鉴定了牛肝脏中55组不饱和甘油磷脂的C=C位置异构体。随后,又通过选择合适的P-B试剂^[74]^,进一步拓展了P-B反应方法,在脂质*sn*-位置和C=C位置产生特异性的离子,从而实现对脂质精细结构解析和准确的相对定量分析。综上所述,光化学衍生联合MS/MS,已经实现了大规模脂质结构表征,能够同时识别C=C位置和*sn*-位置,并且成功区分了人乳腺癌细胞的不同亚型^[74]^。

**图5:**
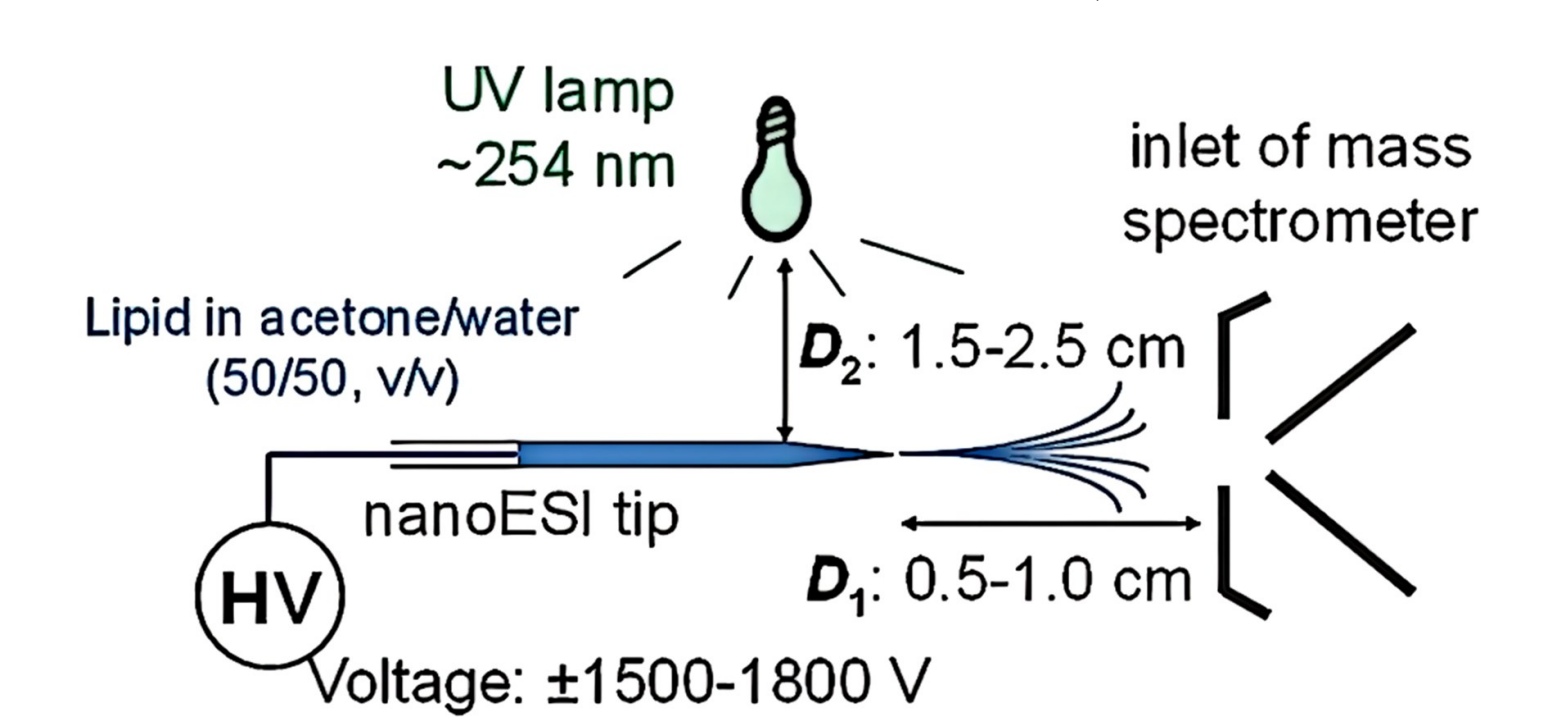
P-B反应与MS在线偶联用于脂质分析的实验设置图^[33]^

P-B-MS/MS反应用于脂质分析的特点是装置简单,无需对MS仪器进行改装。通过从P-B产物中得到的特征碎片离子可以准确鉴定C=C位置异构体,且碎片离子的丰度可进一步用于定量分析。通过优化P-B反应的衍生化方法可实现更广泛的不饱和脂质C=C键定位,如利用探针改变采样方法等^[75]^值得进一步探索。

### 3.2 基于环氧化反应的脂质C=C位置异构体鉴定

Ma和Ouyang等^[76]^在2017年首次将环氧化反应和MS/MS相结合,对FA的C=C异构体进行了结构表征和定量分析。该工作利用低温等离子体(low temperature plasma, LTP)促进脂质C=C键环氧化反应,继而结合MS/MS分析,成功实现了不饱和脂肪酸的结构分析。如图6所示,将不饱和脂肪酸溶解在含1% NH_4_OH的丙酮-水(50∶50, v/v)溶液中,再向该溶液中吹入等离子体,使不饱和脂肪酸的C=C键被环氧化,然后对所得溶液进行ESI-MS和MS/MS分析来鉴定不饱和脂肪酸。但环氧化-MS/MS方法并不适用于分析FA以外的脂质,例如不饱和磷脂。基于此,又在已有的研究基础上进行改进,以乙腈为溶剂,借助氦气将LTP吹入磷脂溶液中,促进脂质中C=C键的环氧化反应,有效拓展了脂质的检测范围^[34]^。同时,利用该方法,还成功分析了牛肝脏的复杂磷脂提取物,鉴定了PC、PE、PA、PG、PI等多种磷脂。间氯过氧基苯甲酸(*m*-CPBA)是一种能够快速、特异性地氧化脂质C=C键的氧化剂。基于此,Li等^[77]^创新性地将*m*-CPBA用于环氧化反应,并与MS/MS联合,实现了对不饱和脂质的C=C键定位。研究表明,在紫外线照射下,安息香能够将O_2_中的氧原子转移形成苯甲酰氧,而酰基过氧自由基能够将含C=C键的碳氢化合物转化为光环氧化产物。基于此原理,Xu等^[78]^首次将光环氧化应用于不饱和脂类的衍生化,并结合CID对不饱和脂类进行鉴定。利用该方法,成功鉴定了小鼠脑组织中5种不饱和脂肪酸的C=C键异构体和5种含有18∶1酰基链的甘油磷脂,还定量分析了小鼠脑、肾、肝和脾等不同组织中的不饱和脂肪酸异构体。此外,Hsu等^[79]^将*m*-CPBA引

发的脂质C=C环氧化衍生反应与LC-MS联用,成功鉴定了人血清中100多种脂质异构体。

**图6:**
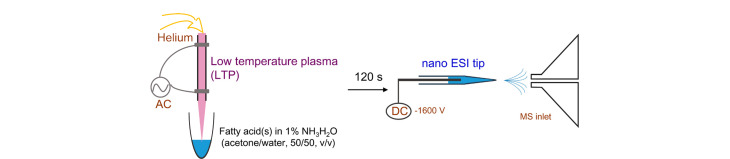
低温等离子体催化不饱和脂肪酸环氧化的实验装置及MS和MS/MS分析^[76]^

环氧化-MS/MS反应用于脂质分析的特点也是无需改装MS即可直接检测。该技术进一步发展的方向是寻找具有高挥发性且特异性强的脂质C=C键环氧化试剂。

### 3.3 基于N-H氮丙啶衍生化的脂质C=C位置异构体鉴定

烯烃的氮杂环丙烷化反应是合成氮丙啶化合物最经典的反应^[80]^。基于此,Yan等^[81]^开发了一种基于氮丙啶的同量异位标记策略,并实现了脂质异构体的鉴定和相对定量分析。首先是利用氮丙啶对脂质中的C=C键进行衍生化,继而通过CID裂解氮丙啶,最后根据特征碎片离子定位不饱和脂质C=C的位置。所形成的氮丙啶中的N-H能够对不同脂质进行标记,从而实现脂质的准确定量,借助该策略,实现了在不使用脂质标准品的情况下对阿尔茨海默病(AD)小鼠血清中17种胆固醇酯异构体的鉴定和定量研究。随后,Yan等^[35]^对非极性甾醇脂质中C=C键进行N-H氮丙啶化,利用CID解离出可定位C=C键的特异性离子,成功鉴定了小鼠前列腺癌组织中的非极性脂质。此外,Chen等^[82]^借助纳升静电喷雾离子化质谱装置,研究了N-Me氮杂环丙烷化反应的中间体。在此基础上,进一步开发了可见光激活的脂质C=C键氮丙啶衍生化方法,并利用CID解离出特征离子,实现了脂质C=C的定位^[83]^。又利用该方法有效实现了小鼠脑缺血过程中磷脂酰胆碱(PC)*sn*-异构体的定量分析(图7)。

**图7:**
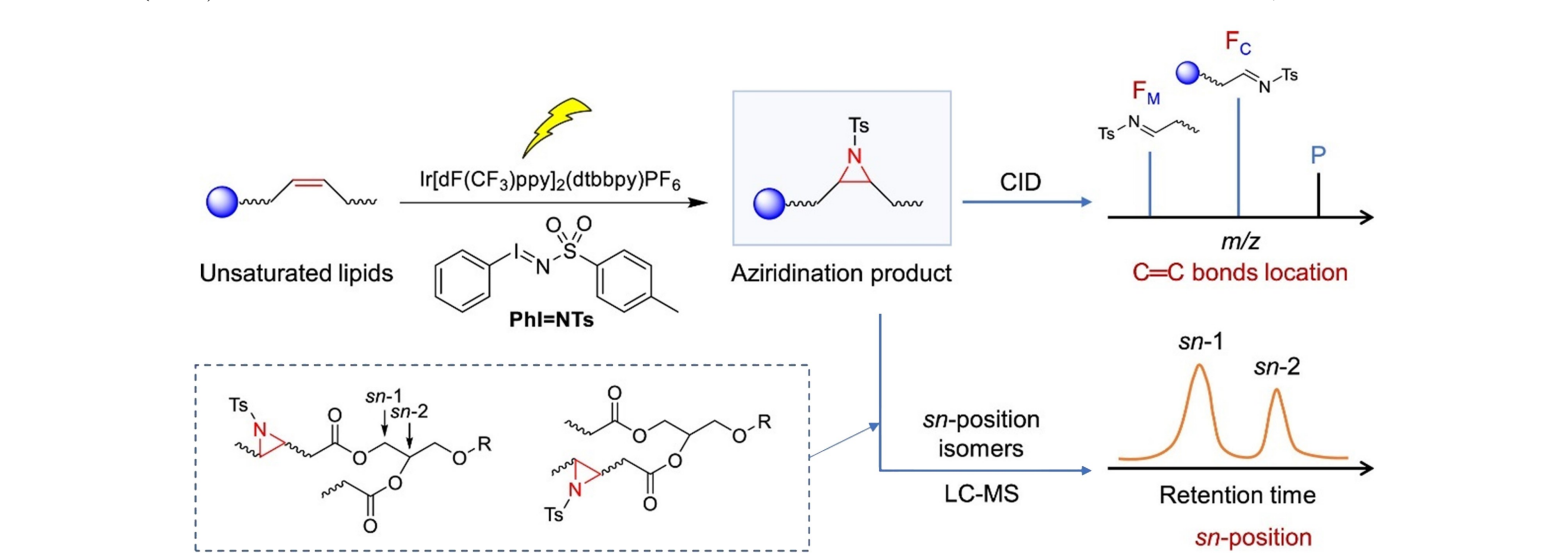
同时分析氮丙啶化衍生化不饱和脂质的C=C键位置和*sn*-位置异构体的策略^[83]^

### 3.4 基于^1^ΔO_2_ ene反应的脂质C=C位置异构体鉴定

^1^O_2_是含有一个空轨道的激发态分子氧,可以与富含电子的双键反应生成氢过氧化物^[84]^。^1^O_2_可与脂质反应生成脂质氢过氧化物^[85]^,经CID处理,脂质氢过氧化物基团发生断键,进而可以确定脂质C=C键位置。基于此,Laskin等^[36]^报道了一种在线光化学衍生方法,即^1^O_2_选择性地与C=C键反应来鉴定脂质异构体;进一步将该方法与基于ESI和nano-DESI的鸟枪法脂质组学技术相结合,成功实现了生物组织提取物的分析和组织切片中异构体脂质的成像(图8)。类似地,Nakagawa等^[86]^借助LC-MS/MS分析了食品样品中被^1^O_2_氧化产生的二十二碳六烯酸氢过氧化物(DHA-OOH),并区分了鲭鱼样本中脂质异构体。

**图8:**
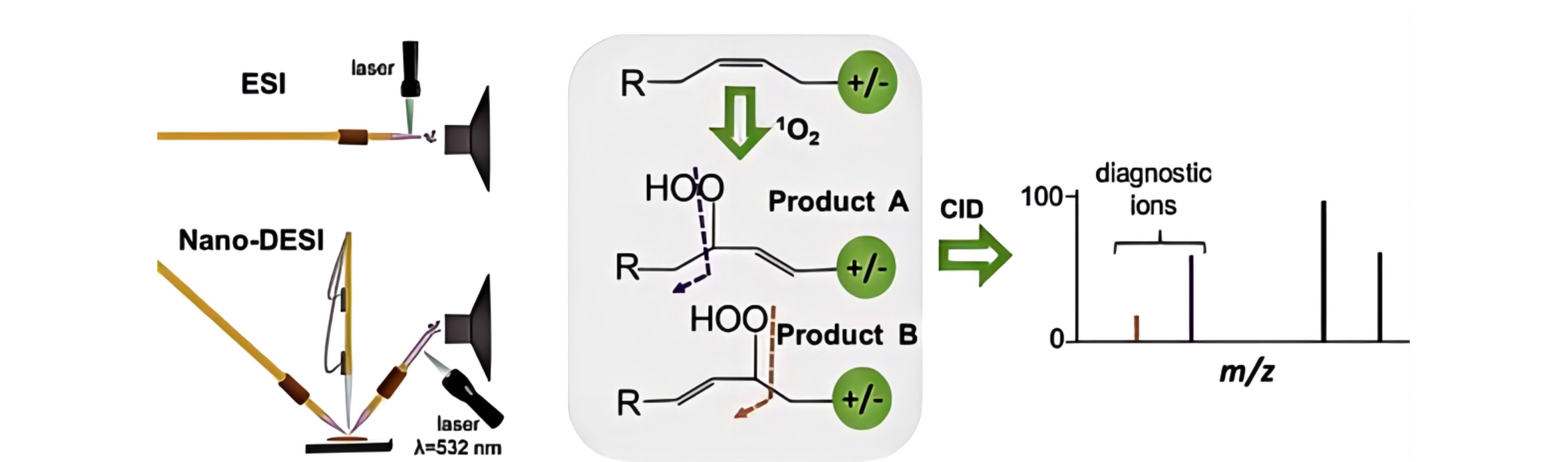
使用单线态氧的光引发反应对脂质进行快速衍生化策略^[36]^

## 4 基于AMS识别脂质其他异构体

不饱和脂质的C=C键位置和*sn*-位置对其生物学功能有很大影响^[87]^。因此,对脂质*sn*-位置识别策略的建立和应用对生物学功能的研究和应用具有重要意义。除了基于AMS的C=C脂质异构体识别,还有一些脂质其他异构体的识别方法。如Blanksby等^[88]^将甘油磷脂的[M+Na]^+^进行CID产生的子离子分离,并进行气相臭氧分解(MS^3^CID-OzID),最后产生的子离子能够反映甘油主链*sn*-位置上酰基取代的特征。在用电喷雾离子化脂质过程中,相关参数如下:毛细管电压为45 V、离子喷雾电压为4 kV、毛细管温度为200 ℃以及管透镜电压为180 V。通过CID/OzID分析成功区分了PC 16∶0/18∶1和PC 18∶1/16∶0的*sn*-位置异构体,这种方法在鉴别来自同一生物的不同组织中异构体成分的相对丰度变化方面具有很大潜力。对于磷脂酰胆碱和鞘磷脂,其胆碱头基可与碳酸根离子结合,在MS/MS中产生*sn*-1特征碎片离子。基于此,Xia等^[89]^通过PC的[M+HCO_3_]^-^在CID中形成*sn*-1特异性碎片离子,成功识别了牛肝脏的极性脂质提取物中82种PC的*sn*-位置异构体。此外,Brodbelt等^[90]^首先使用(三甲基硅基)重氮甲烷(TMSD)将心磷脂(CL)的磷酸基团甲基化,再通过混合高能碰撞活化/紫外光解离(HCD/UVPD)生成CL的特征产物离子,由此区分并定量了CL中的*sn*-立体异构体(图9)。

**图9:**
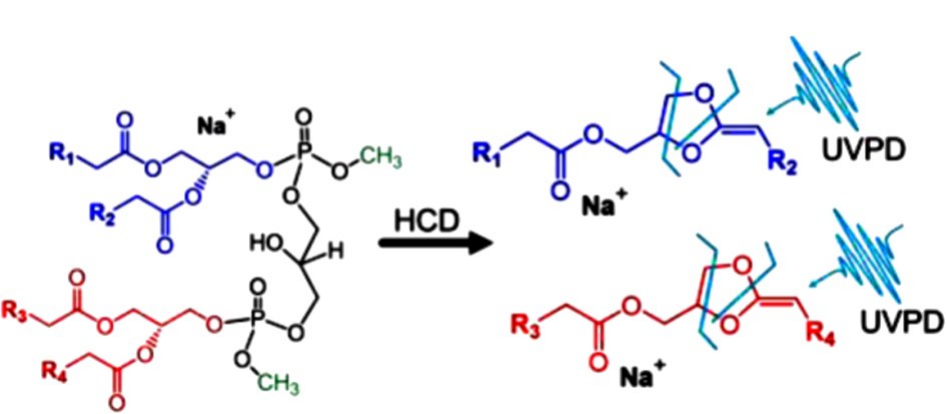
结合TMSD化学衍生和紫外光解离质谱实现对CL结构表征的策略^[90]^

## 5 总结与展望

脂质代谢改变和各种疾病密切相关,脂质组学分析在癌症的诊断、治疗和研究等相关过程中发挥了关键作用。结合脂质检测的快速、高通量及成像定位等研究需求,借助AMS快速检测的优势,人们利用ESI-MS、DESI-MS、化学衍生等方法开展了广泛的癌症脂质代谢研究。该系列研究不仅实现了脂质异构体的鉴定和相对定量分析,还可以结合各种化学衍生法确定脂质异构体的C=C位置,有助于脂质结构异构体分析。尽管AMS在脂质组学领域取得了重大进展,但是在复杂脂质的全面鉴定和结构表征方面仍然具有诸多挑战性。由于脂质多样异构体的存在,除了大量定位C=C键的研究外,还需要研究者在识别*sn*-位置、支链异构体和脂质空间分布方面进一步深入研究。

AMS由于其快速、高灵敏度和高化学特异性的分析优势,将为脂质精细结构鉴定、相对含量分析、生物过程中的动态转化以及与其他生物分子之间的相互作用研究等提供最有效的研究手段。这些特性也使人们对AMS在基于脂质的高通量代谢研究、疾病潜在生物标志物发现、生物组织成像等方面的应用寄予很高的期望。
